# Thoughts on the usefulness of a new scoring system for heart failure

**DOI:** 10.1007/s12471-022-01715-6

**Published:** 2022-08-12

**Authors:** P. Meregalli

**Affiliations:** Department of Cardiology, Amsterdam UMC, location AMC, Amsterdam, The Netherlands

Heart failure (HF) is a highly prevalent and severe cardiac syndrome with a high impact on the patient’s quality and quantity of life. Due to population ageing and the introduction of enhanced cardiovascular techniques, resulting in improved survival following acute cardiac events, the incidence of HF is increasing over time. These developments have a major influence on the organisation and the cost of outpatient care.

In an ideal situation, specialised care should be available whenever a HF patient deteriorates, while stable patients on optimal medical treatment could be safely discharged from the outpatient clinic [1]. There is debate as to the best time for referral to the general practitioner (GP) and which criteria should be used for objective selection. A validated protocol is lacking.

In their present study, Gingele et al. aimed to assess the feasibility of a new scoring system, called the Maastricht Instability Score—Heart Failure (MIS-HF) questionnaire, as a tool for identifying which subgroup of HF patients are stable enough to be safely referred to the GP [2]. They sought to investigate whether the prognosis of the patients receiving first-line treatment remained comparable to that of those seen at the HF units.

This analysis was done in a retrospective manner and with obvious limitations, as pointed out by the authors themselves. The MIS-HF tool consists of a comprehensive questionnaire comprising 33 items, which include not only clinical data on the severity of HF, but also levels of the biomarker N‑type pro-brain natriuretic peptide and other serum parameters as well as electrocardiographic data.

The decision as to how many points were given per item was arbitrary, but the authors state that they wished to remain on the conservative side. They advise that patients with a low score (total of 0–1 or 2) should be referred for primary care, while patients with a higher score should remain under specialised care.

Undoubtedly, patients with a total score of 0 can be considered stable. The same can very likely be said for scores of 1 or 2. It is questionable whether New York Heart Association (NYHA) class III patients can be regarded as stable. According to the MIS-HF score, as proposed by Gingele et al., NYHA class III is assigned ‘only’ 1 point, whereas the risk of (re)hospitalisation in these patients is real. Another issue with the NYHA classification remains its limited objectivity and the significant overlap between classes II and III. As such, the NYHA classification is not sufficient.

Otherwise, the study is well written and provides many aspects for further investigation. First of all, the authors demonstrate that the clinical implementation of such a questionnaire is safe and achievable in daily practice. There was no significant difference in the composite endpoint between patients with a low MIS-HF score treated in primary versus secondary care.

This is encouraging for further research in the same direction. Nevertheless, almost 6% of the patients with a low MIS-HF score died and 7% needed hospitalisation within 1 year of follow-up. It is of crucial importance that the patients can easily re-access secondary care if they develop symptoms and signs of deterioration. We are not informed about how often the patients in both groups were actually seen for regular or urgent visits, but the median time to first outpatient clinic visit was 168 days, which is fairly long.

Before implementing such a scoring system hospitals should, therefore, be able to re-admit deteriorating patients at any time. In my opinion, sharing the same electronic patient dossier is an essential step toward multidisciplinary care. Implementation of real-time access to patients’ data for all involved care givers is mandatory. With this premise, it could be possible to firstly alternate regular visits to the specialist and the GP practice for at least 1 year before taking the definitive decision for referral to the GP. In this manner, patient compliance will possibly increase, due to the fact that they can adapt to this new situation. The psychological and social conditions definitely play a role in the way follow-up is organised. A positive feature is that both social support and mental status are included in the questionnaire.

Unfortunately, no detailed information is given about medical treatment. We can assume that all patients were on optimal medical treatment at the time of enrolment (2015–2018). This aspect should be taken into consideration because new HF medication (sacubitril/valsartan, sodium-glucose cotransporter 2 inhibitors) is increasingly implemented in daily practice [3, 4]. The initiation and the uptitration of specialised HF medication is usually performed in secondary care, which may delay referral to the GP [5].

Finally, it is shown that the MIS-HF questionnaire is an objective and useful tool for the referral process from secondary to primary care, but one may wonder whether it is also suitable for referral in the other direction. The use of laboratory and ECG data makes it quite complex. One might consider a two-step protocol for the GP: a first step aimed at identifying the presence of HF-related symptoms and a second step for further assessment of serum, electrocardiographic and echocardiographic parameters. During this second phase, collaboration with the cardiologist and/or the HF nurse should be encouraged.
